# Xenotransplantation of Human Cardiomyocyte Progenitor Cells Does Not Improve Cardiac Function in a Porcine Model of Chronic Ischemic Heart Failure. Results from a Randomized, Blinded, Placebo Controlled Trial

**DOI:** 10.1371/journal.pone.0143953

**Published:** 2015-12-17

**Authors:** Sanne J. Jansen of Lorkeers, Johannes M. I. H. Gho, Stefan Koudstaal, Gerardus P. J. van Hout, Peter Paul M. Zwetsloot, Joep W. M. van Oorschot, Esther C. M. van Eeuwijk, Tim Leiner, Imo E. Hoefer, Marie-José Goumans, Pieter A. Doevendans, Joost P. G. Sluijter, Steven A. J. Chamuleau

**Affiliations:** 1 Department of Cardiology, University Medical Center Utrecht, Utrecht, the Netherlands; 2 ICIN - Netherlands Heart Institute, Utrecht, the Netherlands; 3 Experimental Cardiology Laboratory, University Medical Center Utrecht, the Netherlands; 4 Department of Radiology, University Medical Center Utrecht, Utrecht, the Netherlands; 5 Department of Molecular Cell Biology, Leiden University Medical Center, Leiden, the Netherlands; Georgia Regents University, UNITED STATES

## Abstract

**Background:**

Recently cardiomyocyte progenitor cells (CMPCs) were successfully isolated from fetal and adult human hearts. Direct intramyocardial injection of human CMPCs (hCMPCs) in experimental mouse models of acute myocardial infarction significantly improved cardiac function compared to controls.

**Aim:**

Here, our aim was to investigate whether xenotransplantation via intracoronary infusion of fetal hCMPCs in a pig model of chronic myocardial infarction is safe and efficacious, in view of translation purposes.

**Methods & Results:**

We performed a randomized, blinded, placebo controlled trial. Four weeks after ischemia/reperfusion injury by 90 minutes of percutaneous left anterior descending artery occlusion, pigs (n = 16, 68.5 ± 5.4 kg) received intracoronary infusion of 10 million fetal hCMPCs or placebo. All animals were immunosuppressed by cyclosporin (CsA). Four weeks after infusion, endpoint analysis by MRI displayed no difference in left ventricular ejection fraction, left ventricular end diastolic and left ventricular end systolic volumes between both groups. Serial pressure volume (PV-)loop and echocardiography showed no differences in functional parameters between groups at any timepoint. Infarct size at follow-up, measured by late gadolinium enhancement MRI showed no difference between groups. Intracoronary pressure and flow measurements showed no signs of coronary obstruction 30 minutes after cell infusion. No premature death occurred in cell treated animals.

**Conclusion:**

Xenotransplantation via intracoronary infusion of hCMPCs is feasible and safe, but not associated with improved left ventricular performance and infarct size compared to placebo in a porcine model of chronic myocardial infarction.

## Introduction

The heart has regenerative capacity as it harbours a pool of cardiac stem cells.[1] However, this is clearly not sufficient to repair the damage caused by myocardial infarction (MI) to prevent the development of heart failure. The number of stem cells available might just be too little. *Ex vivo* expansion and reapplication of cardiac stem cells to the injured heart was proposed, however isolation of these cardiac stem cells remains challenging. Our lab succeeded in isolating fetal and adult cardiomyocyte progenitor cells (CMPC) from mouse and human hearts based on the stem cell antigen Sca-1.[2] CMPCs can successfully been differentiated in cardiomyocytes, endothelial cells and smooth muscle cells *in vitro*. [2–4] These hCMPC-derived cardiomyocytes have functional gap junctions, enabling metabolic and electrical coupling of cells.[4] Intra-myocardial injection of hCMPCs in a mouse model of acute MI led to engraftment of 3,5% of cells, differentiation towards coupled cardiomyocytes, increased vascular density, and to improved cardiac function. [5] These promising results led to the current large animal study, as a next step towards potential clinical application of hCMPCs. Since the greatest burden of ischemic heart disease is based on chronic ischemic heart failure, hCMPCs were tested in a chronic disease model.

In the current study, fetal hCMPCs were intracoronary administered in a porcine model of chronic ischemia/reperfusion injury (I/R). We hypothesized that this strategy is safe, improves left ventricular performance and reduces infarct size compared to placebo.

## Methods

### Experimental design

All animal experiments were executed in conformance with the ‘Guide for the Care and Use of Laboratory Animals’. The experiment was evaluated and approved by the Animal Experiments Committee of the Utrecht University, the Netherlands (permit number 2012.II.09.145).

**Fig 1:**
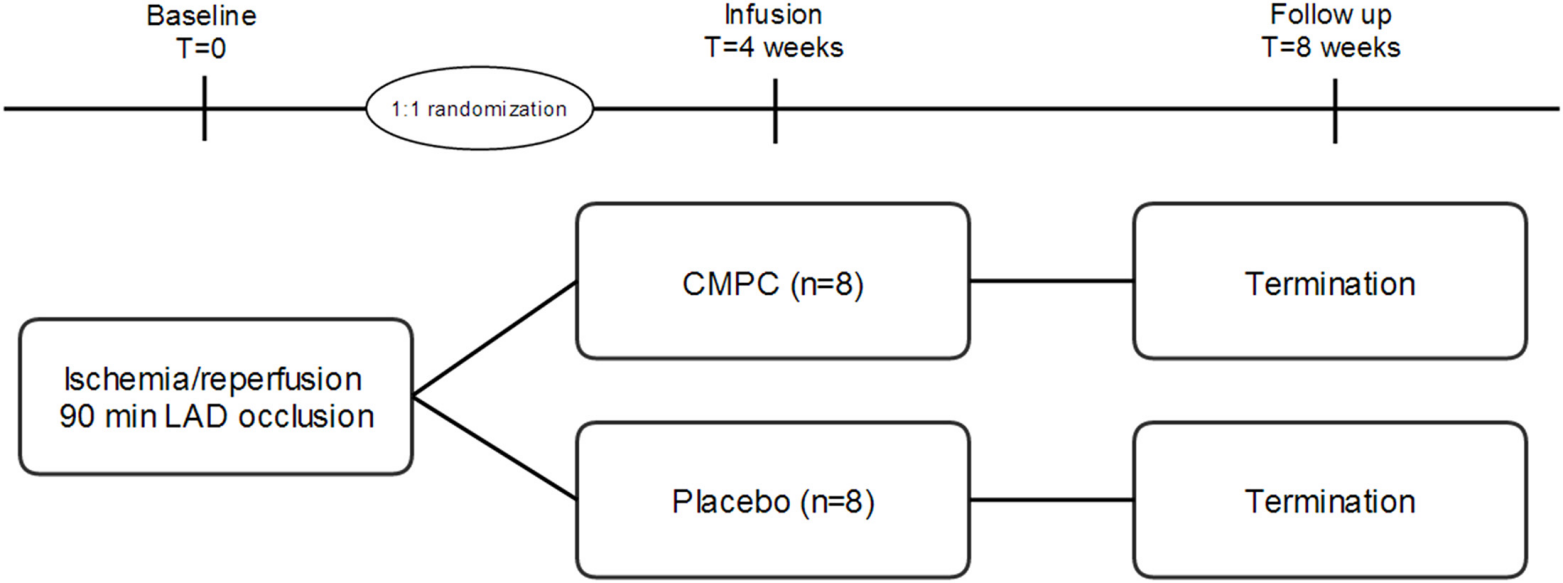
Study protocol.

Animals were subjected to I/R, randomized to fetal hCMPC or placebo infusion 4 weeks later and endpoint analyses were performed 4 weeks after stem cell injection (see study protocol, Fig 1). All animals were immunosuppressed by Cyclosporin A (CsA) to facilitate xenogeneic cell treatment. Based on a power calculation (estimated effect 7.5% [6], standard deviation of 5%, a power of 0.9 and alpha of 0.05) 8 pigs per group were needed. Animals that died before endpoint analysis were supplemented.

Animals were randomized after surviving the initial I/R, using a computer based random order generator. Cell and placebo infusion as well as data analysis were performed in a blinded fashion (investigators, technicians and animal caretakers). Deblinding was performed after collecting and analysing all data.

The primary endpoint of this study was defined as left ventricular ejection fraction (EF) at the end of follow-up, measured by magnetic resonance imaging (MRI). Secondary endpoints were left ventricular end diastolic volume and left ventricular end systolic volume (EDV and ESV) measured by MRI, infarct size measured by *ex vivo* gross macroscopy after incubation with triphenyltetrazolium chloride (TTC) and late gadolinium enhancement (LGE) MRI, functional parameters serially measured by pressure volume (PV-)loop and echocardiography, coronary microvascular function by intracoronary pressure- and flow measurements and vascular density and fibrosis on histology.

### Cell isolation

Cells were isolated from fetal human heart tissue (derived after elective abortion with informed consent) and cultured as described before. [2] Shortly, tissue was minced, incubated with collagenase and grinded through a cell strainer. Cells were incubated with anti-Sca-1 microbeads and separated using a MiniMACS magnet (Miltenyi Biotec, Leiden, the Netherlands). hCMPCs were collected and dissolved in growth medium containing endothelial growth medium (EGM-2, Cambrex, CC-4176), FBS, penicillin/streptomycin, non-essential amino acids and bFGF. After attachment of the cells, cells were split after +/- 3 days at 80–90% confluence in a 1:6 fashion. All pigs received hCMPCs of passage 5–7 from the same donor.

### Cell characteristics

Fetal hCMPCs are characterized by expression of CD105, CD31, Sca-1 and c-kit and absence of expression of CD45, CD14,CD34 and CD133.[2] Further cell characteristics are provided in Table 1. CMPCs from fetal and adult human hearts showed differentiation into spontaneously beating and electrically coupled cardiomyocytes after stimulation with 5-azacytidine and TGFβ.[4] Differentiation into endothelial cells and smooth muscle cells can be achieved by exposure to vascular endothelial growth factor (VEGF).[2–4] CMPCs in culture are presented in S1 Fig.

**Table 1:** CMPC characteristics.

Passage	5–7
Cell size	14–23 μm
Population doubling time	8.25 hours
Viability	96%
Fetal age	12 weeks

### Animal experiment

A comprehensive description of the protocol is also available at http://www.jove.com/video/51269. [7] Female landrace specific pathogen free pigs (n = 19) (van Beek SPF Varkensfokkerij B.V. Lelystad, the Netherlands), weighing 68.5 ± 5.4 kg (Upper and lower limit 60.6–82.0 kg) at baseline, were pre-treated with amiodaron for 10 days (1200 mg/day for 10 days, 800 mg/day maintenance), clopidogrel for 3 days (75 mg/day) and acetylsalicylic acid for 1 day (320 mg loading dose, 80 mg/day maintenance) and a fentanyl patch (25μg/h) for 1 day. One day before cell or placebo delivery, cyclosporin (CsA) was started. Based on clinical organ transplantation protocol,[8] pigs received a loading dose of 800 mg, then 400 mg b.i.d. for 1 week and 200 mg b.i.d for the remaining 3 weeks by oral administration (Neoral drink, 100mg/ml, Novartis Pharma bv). At the day of surgery, one dosage was i.v. infused as 200 mg in 100mL over 2 hours (Sandimmune 50mg/ml, Novartis Pharma bv). All medication, except for the fentanyl patch, was continued until the end of follow up. Pigs received fiber rich pellets (Abdiets animal nutrition, product 2755, Woerden, The Netherlands) twice a day and water was available ad libitum. Animals were kept fasted the day of surgery (except for medication).

Anaesthesia was obtained by intramuscular injection of 10 mg/kg ketamine, 0.4 mg/kg midazolam and 0.5 mg/kg atropine in the cage. Pigs were intubated and transported to the operating theatre. Maintenance anaesthesia consisted continues infusion of 0.5 mg/kg/h midazolam, 2.5 μg/kg/h sufentanyl and 0.1 mg/kg/h pancuronium. Other perioperative medication consisted of 300 mg amiodaron, amoxicillin + clavulanic acid 750/75 mg and heparin (100 IE/kg after positioning the sheaths and 50 IE/kg every 2 hours). Pigs were mechanically ventilated with a positive pressure ventilator with FiO2 0.5, 10ml/kg tidal volume and a frequency of 12/minute under continuous capnography. Arterial access was achieved by cannulating the internal carotid artery with an 8F sheath. Venous access was achieved by cannulating the jugular vein with a 9F sheath. An additional arterial line was inserted in a small peripheral artery in the hind limb for continuous stable arterial pressure registration.

### Echocardiography

Pigs were positioned in the right lateral position. Parasternal short axis images were obtained at 3 levels (mitral valve, papillary muscle and apex) during 5 beats per level (iE33 ultrasound device Philips, Eindhoven, The Netherlands). Short axis images were analysed offline using Xcelera R2.L1 (Philips Healthcare, Best, The Netherlands) and fractional area shortening (FAS), fractional shortening (FS) and septal wall thickening (WTsept) at the level of the mitral valve, papillary muscle and apex (mitral, pap, apex respectively) were calculated. Because of apical dilatation of the left ventricle after infarct, the modified Simpsons rule was not applicable and no reliable volumes could be calculated.

#### Pressure volume loop measurement

Admittance based PV-loop measurements were performed as recently described. [9] Cardiac output (CO) was measured three times by thermodilution and stroke volume was calculated out of three measurements. The 7F tetra-polar admittance catheter (7.0 VSL Pigtail/no lumen, Transonic Scisense, London, Canada) was inserted into the left ventricle through the arterial sheath under fluoroscopic guidance. Inferior caval vein occlusion was performed using an 8F Fogarty occlusion catheter (62080814F, Edwards Lifesciences). All measurements were performed during apnea. Data were offline analysed using Iworx analysis software (Labscribe V2.0).

### Intracoronary pressure and flow velocity assessment

Intracoronary pressure and flow were assessed by positioning a Combowire in the left anterior descending artery (LAD) and the left circumflex coronary artery (LCX) acting as a control subsequently. Intracoronary pressure and flow measurements, together with arterial pressure and ECG, were recorded using the ComboMap system (Volcano Corporation). An intracoronary bolus of 200 μg nitroglycerin was administered to prevent coronary spasms. Three successive measurements were recorded in rest and during hyperaemia, achieved by intracoronary administration of 60 μg adenosine. Data were analysed offline, using AMC Volcano Studymanager (versus 6.0, Borland Software Corporation and Delphi versus 2010, Embarcadero, San Francisco, CA, USA).

### Ischemia/reperfusion injury

After baseline measurements, the diameter of the LAD, distal to the second diagonal branch (D2) was measured in anterior-posterior and left anterior oblique 30° view. Before positioning the balloon, an intracardiac defibrillation catheter was placed in the right ventricle. A PCI-balloon catheter with a suitable diameter was positioned distal from the D2 and inflated for 90 minutes. In case of ventricular arrhythmias, chest compressions were started immediately, amiodaron 300mg was infused intravenously and pigs were defibrillated intracardiac with 50J. In case of multiple unsuccessful shocks, defibrillation was switched to transthoracic defibrillation with 150-200J.

### Cell infusion

Ten million cells were resuspended in 10mL of phosphate buffered saline (PBS), one hour before intracoronary infusion. Cell suspension or placebo (10mL of PBS) was handed to the animal technician in a covered sterile syringe so that the personnel at the operating theatre remained blinded for the treatment allocation. An over-the-wire PCI balloon catheter (Apex, Boston Scientific), was positioned distal to the D2, according to the position of the PCI catheter during infarct creation. The balloon was inflated until pressure matched the coronary diameter. 10mL of cell suspension or placebo was infused in three sessions consisting of 30 seconds infusion of 3.3mL during 2 minutes balloon occlusion followed by 3 minutes of reperfusion. Intracoronary pressure and flow measurements were repeated, 30 minutes after the last reperfusion period, to detect possible flow restrictions caused by cell or placebo infusion.

### MRI

At the end of follow up, cardiac MRI and all other functional measurements were performed. All MR studies were performed on a 3T MRI scanner (Achieva TX, software release 3.2; Philips Healthcare, Best, The Netherlands). Both cine images and LGE images in short axis and a two-chamber long axis orientations were obtained under continuous anesthesia and mechanical ventilation. LGE images were obtained at least 15 minutes after injection of 0.2 ml/kg gadobutrol (Gadovist, Bayer Healthcare). Offline imaging analysis for functional measurements was performed in Qmass MR 7.4 enterprise solutions (Medis medical imaging systems BV, Leiden) and by Segment version v1.9R 3293 (Medviso AB, Lund, Sweden) for infarct size. Infarct size at the end of follow-up was analysed based on the 2SD method.

### Infarct size

Animals were sacrificed by exsanguination under general anesthesia. The hearts were excised, washed and cooled with running tap water and the LV was then cut into 5 equal slices from apex to base. Slices were incubated in 1% TTC (Sigma-Aldrich Chemicals, Zwijndrecht, the Netherlands) in 37°C 0.9%NaCl for 15 min to discriminate infarcted tissue from viable myocardium. The infarcted area was calculated as percentage of the left ventricle.

### Histology

Sections of infarct zone, border zone and remote area of all pigs were isolated and fixated in 4% formalin for at least 1 week and then embedded in paraffin. Sections of 5 μm were stained for endothelial cells by lectin (Lectin fom Bandeiraea simplicifolia, Sigma Aldrich L5391) and for fibrosis by picrosirius red (Sirius red F3B, BDH and picrin acid, Boom. Cat. 12388).

For vascular density, 5 random pictures per slide with 40x magnification were taken from the border zone area. The number of vessels was counted manually by two independent observers (S.J. and J.G.) and the absolute number of lectin positive vessels per field were averaged. For assessment of fibrosis, 3 random pictures per slide (slides for infarct zone, border zone and remote area) were taken using polarized light during one session. Pictures were analysed in Cell^p (version 5.0 Olympus) for mean grey value and percentage of fibrotic area by using the same settings for all slides.

### Cyclosporin assay

The effect of CsA on the cells was tested by a migration assay, sprouting matrigel assay and by testing cellular growth factor release.

Based on the pig serum levels of CsA, in vitro assays were executed to analyze performance of CMPCs in presence of CsA. CMPCs were maintained in 0.1% gelatin coated plates with standard CMPC culture medium. For all in vitro experiments low passages were used (passage 8–15). For all assays, standard culture medium was used as a negative control. Three different concentrations of CsA were used as experimental conditions (50 ng/mL, 150 ng/mL and 300 ng/mL). See S1 File for detailed description of the methods.

### QPCR

Genomic DNA was isolated from 1cm^3^ cryosections of cardiac tissue from the borderzone area using the Genomed Jetquick tissue DNA spin kit (cat no 450250) for each CMPC treated pig. Tissue of a PBS treated pig was also included in the experiment as a control. Furthermore the CMPCs used in our study were analysed as a positive control for human DNA. Samples were analyzed for the presence of human (HLA-DMA) and pig (Pig Gapdh) genomic DNA using the following primers:

HLA-DMA fwd, 5’-TACAAACCTCAGCTACCTTCGTGGC-3

HLA-DMA rev, 5’-AACCCAGCTGACTCTGGGTGG-3’

Pig Gapdh fwd, 5’-CCCCCTCAGATTTGGCCGCA-3’

Pig Gapdh rev, 5’-CACGGGGGCCACTCACCAT-3’

After DNA isolation, 250 ng DNA per sample was precipitated, diluted in H2O up to 50 μl and preamplified for 12 cyles using he SsoAdvanced PreAmp Supermix (Biorad 172–5160).

QPCR was performed by adding 9.8 μl SYBR GREEN and 0.2 μl primer (forward and reverse, HLA-DMA and Gapdh) to 10 μl preAMP mix containing 50 ng DNA (denaturation at 95°C; annealing at 61°C; and extension at 72°C). After quantitative PCR, 5 μl of the amplified PCR samples plus 1 μl loading dye were loaded on a 2% agarose gel (5 gram agarose, 250ml Tris-acetate-EDTA buffer).

### Statistical analysis

Data are reported as mean ± standard deviation. Comparisons of serial measurements over time within one group or for all animals combined were performed by paired t-tests. Comparisons between both groups were made by independent t-tests. Differences between conditions in cyclosporin assays were tested by one-way ANOVA.

The statistical software used is IBM SPSS statistics, version 20.0 (IBM Corporation, Armonk, NY, USA).

## Results

A total of 19 pigs were used in this study. In 9 pigs VF occurred during occlusion, of which 8 converted successfully by defibrillation and 1 to 3 intravenous boluses of 150 mg amiodaron infusion. One animal could not be safed by these efforts, despite an additional 0,5 mg adrenaline. A total of three pigs died; one pig died of VF during LAD occlusion, one pig died by periprocedural complications before cell/placebo infusion and one animal died one day after placebo infusion by unknown cause. All other 16 animals were included in this study.

### Safety

Two hours after cell infusion during short coronary occlusion, high sensitive troponin I (TnI) levels were 545 ± 460 ng/L n = 8 after cell infusion and 623 ± 764 ng/L n = 8 after placebo infusion. In comparison, mean TnI levels 3.5 hours after the start of 90 minutes balloon occlusion, were 1147822 ± 584559 ng/L (n = 16), confirming the induction of a large MI.

Intracoronary pressure and flow measurement (see below) showed no evidence for intracoronary embolization or obstruction in CMPC treated animals 30 minutes after infusion. None of the CMPC treated animals died by arrhythmias, or any other cause.

### Left ventricular performance

#### MRI

**Fig 2:**
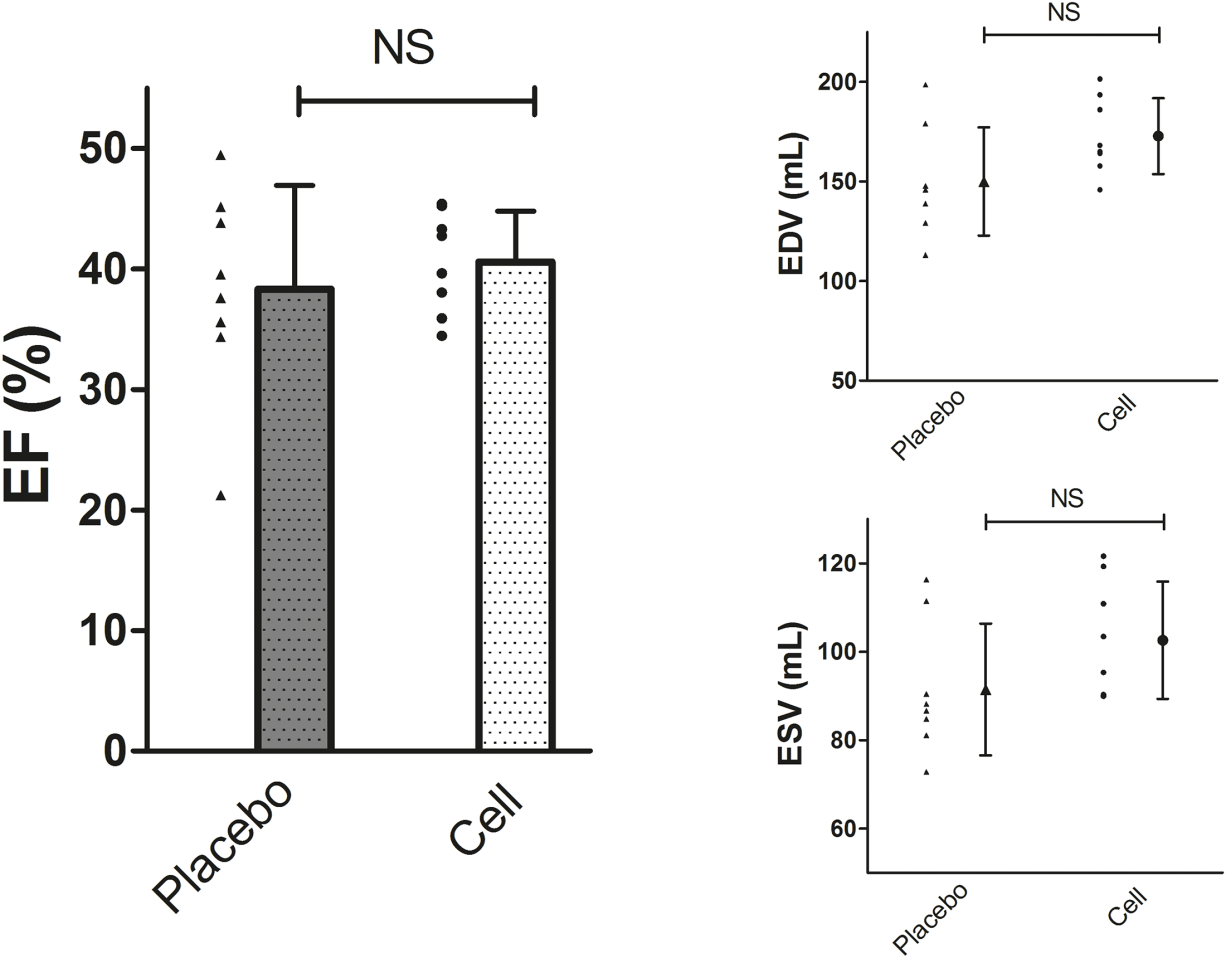
Functional measurements by MRI of cell- and placebo treated animals at follow up. Individual data and mean with SD, n = 8 per group. EF Left ventricular ejection fraction, EDV End diastolic volume, ESV End systolic volume.

At the end of follow-up EF, EDV and ESV did not differ between groups. EF in CMPC treated animals was 40.6 ± 4.2% versus 38.3 ± 8.6% in placebo animals (p = 0.52). EDV was 172.7 ± 19.0 ml in CMPC treated animals and 150.0 ± 27.1 ml in placebo (p = 0.07). ESV was 102.6 ± 13.3 ml in CMPC treated and 91.5 ± 14.9 ml in placebo animals (p = 0.14). (Fig 2)

#### PV-loop

Serial functional measurements were performed by PV-loop. Because of technical failure, we could not complete data analysis of all animals at all time points. For EF, ESV and EDV we could include 7 out of 8 cell treated animals and all 8 placebo animals. For end systolic pressures volume relationship (ESPVR), end diastolic pressure volume relationship (EDPVR) and V0, we could include 6 animals per group.

**Fig 3:**
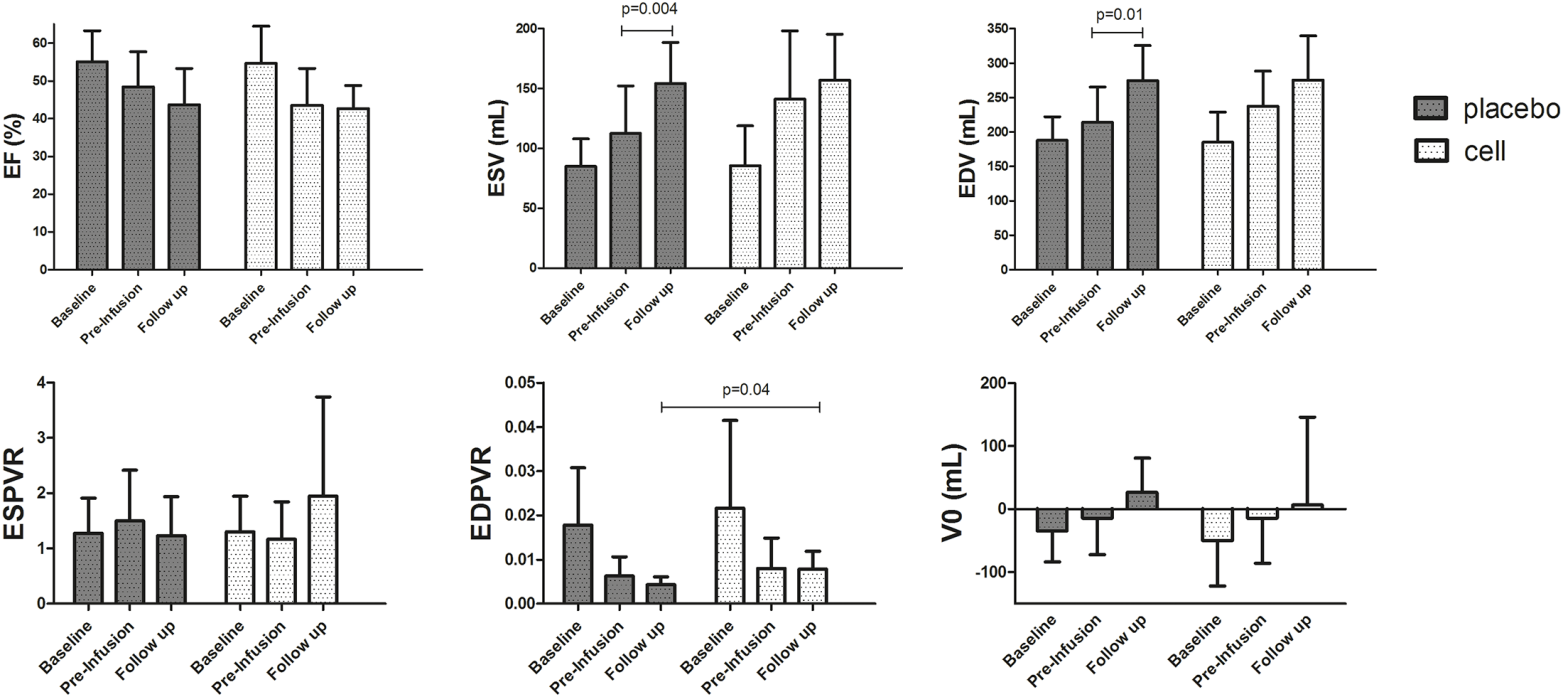
Functional outcome measurements by PV loop. Mean and SD for PVloop parameters. EDV End diastolic volume, ESV End systolic volume, EF Left ventricular ejection fraction, ESPVR End systolic pressures volume relationship, EDPVR end diastolic pressure volume relationship. Placebo n = 8, cell n = 7 for ESV, EDV and EF, n = 6 per group for ESPVR, EDPVR and V0.

Induction of MI caused a significant decrease in EF over 4 weeks (-8.7 ± 11.6% from 54.8 ± 8.7 to 46.1 ± 9.5 p = 0.01) and an increase in ESV (+40.6 ± 62.3 ml from 85.3 ± 27.1 to 125.9 ± 49.1 p = 0.02), and trend towards an increased EDV (+38.1 ± 73.6 ml, from 186.7 ± 37.6 ml to 224.7 ± 50.9 p = 0.07), confirming successful creation of a chronic MI model. No significant differences between both groups were observed at pre-infusion time point (EF p = 0.34, EDV p = 0.40, ESV p = 0.27). (Fig 3)

For all parameters (EDV, ESV, EF, ESPVR, EDPVR and V0), no differences between both groups were found at any time point except for EDPVR at follow up. EDPVR was 0.004 ± 0.002 for cell treated animals and 0.008 ± 0.004 for placebo animals (p = 0.04). Visual appearing trends in functional outcomes are observed in Fig 3 (and S2 Table). However, EF did not increase after cell or placebo infusion (p = 0.85 and p = 0.17 respectively) EDV and ESV did not change after cell treatment (p = 0.25 and p = 0.58 respectively), but both significantly increased after placebo infusion (p = 0.01 and p = 0.004), suggesting further deterioration after placebo infusion compared to cell treatment. No statistical significant differences in ΔEDV and ΔESV were found between cell treated animals and placebo animals.

#### Echocardiography

S1 and S2 Videos show representative long and short axis echocardiography images.

WTsept and FAS at the apical level significantly decreased after MI (WTsept -0.34 ± 0.23 p < 0.001, FAS -0.08 ± 0.11 p = 0.02), confirming successful apical infarct creation. A trend towards decrease of FS at the apical level was also observed (-0.50 ± 0.11 p = 0.08) in both groups.

**Fig 4:**
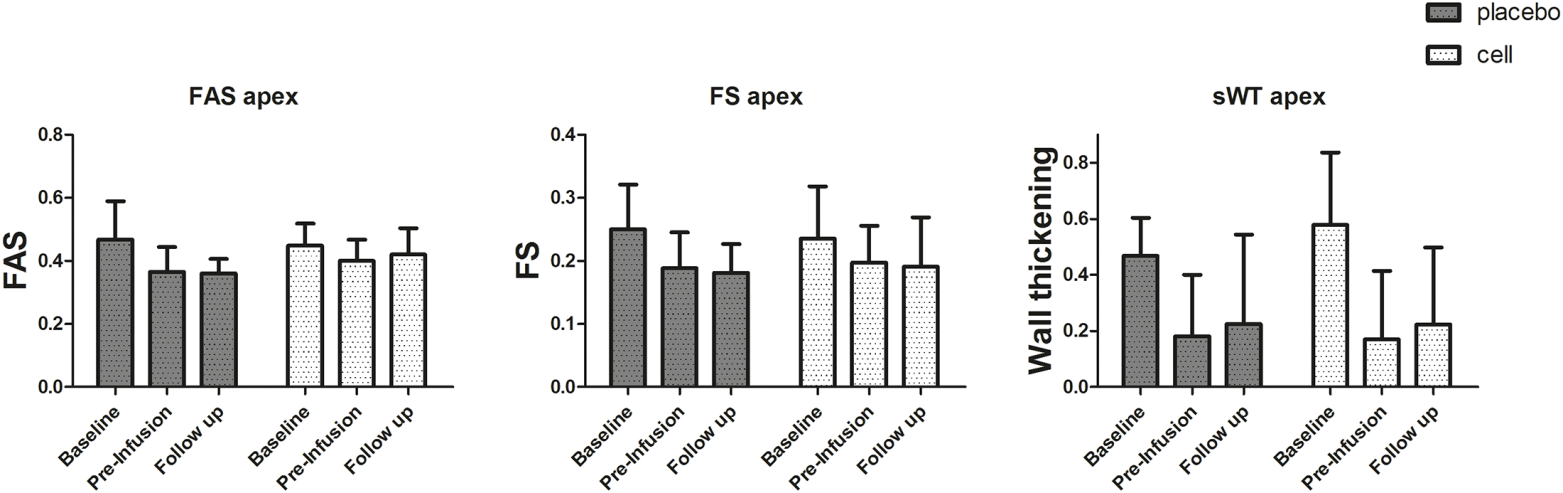
Functional measurements by echocardiography. Mean and SD, n = 8 per group. FAS Fractional area shortening, FS Fractional shortening, sWT Septal wall thickening at the level of the apex.

For FAS, FS and WTsept, no statistical significant differences were identified between groups at any time point. A trend was observed for a higher mean FAS (mean of three levels) at follow up in cell treated animals compared to placebo animals (cell treated 0.49 ± 0.06 placebo 0.44 ± 0.04 p = 0.08). Next, no differences in decrease or increase of any other echocardiographic parameter after hCMPC or placebo infusion was observed. (Fig 4 and S3 Table)

### Intracoronary pressure and flow

Hyperemic microvascular resistance is a parameter for assessing the microvascular bed and is calculated as the ratio of intracoronary pressure (Pd) and hyperemic average peak flow velocity (pAPV). It is known from previous studies that HMR increases after I/R, accompanied by decreased pAPV and a constant Pd.[10] At the time point of cell infusion, measurements in one animal failed because of technical problems.

HMR in the LAD region significantly increased after I/R as expected (p = 0.03), Pd in the LAD significantly increased (p = 0.03) and pAPV in the LAD tended towards a decrease of (p = 0.10). All parameters in the control region remained stable over time. Thirty minutes after cell or placebo infusion, HMR was significantly decreased in all animals (2.7 ± 1.0 mmHg/cm/s pre-infusion, 2.2 ± 0.6 mmHg/cm/s post-infusion, p = <0.001), without any differences between hCMPC and placebo treated animals (ΔHMR -0.50 ± 0.48 mmHg/cm/s for hCMPC treated, -0.56 ± 0.66 mmHg/cm/s for placebo treated animals, p = 0.21). Decrease in HMR was accompanied by an increase in pAPV (41.5 ± 11.0 cm/s pre-infusion, 48.9 ± 11.7 post-infusion, p = 0.01), without differences between groups (p = 0.7), both advocating against intracoronary (micro-)embolization or obstruction by cell infusion. Decreased HMR and increased pAPV can be explained as a hyperemic response after short ischemia during infusion. Neither cell-, nor placebo infusion caused a change in microvascular parameters over time.

### Infarct size

**Fig 5:**
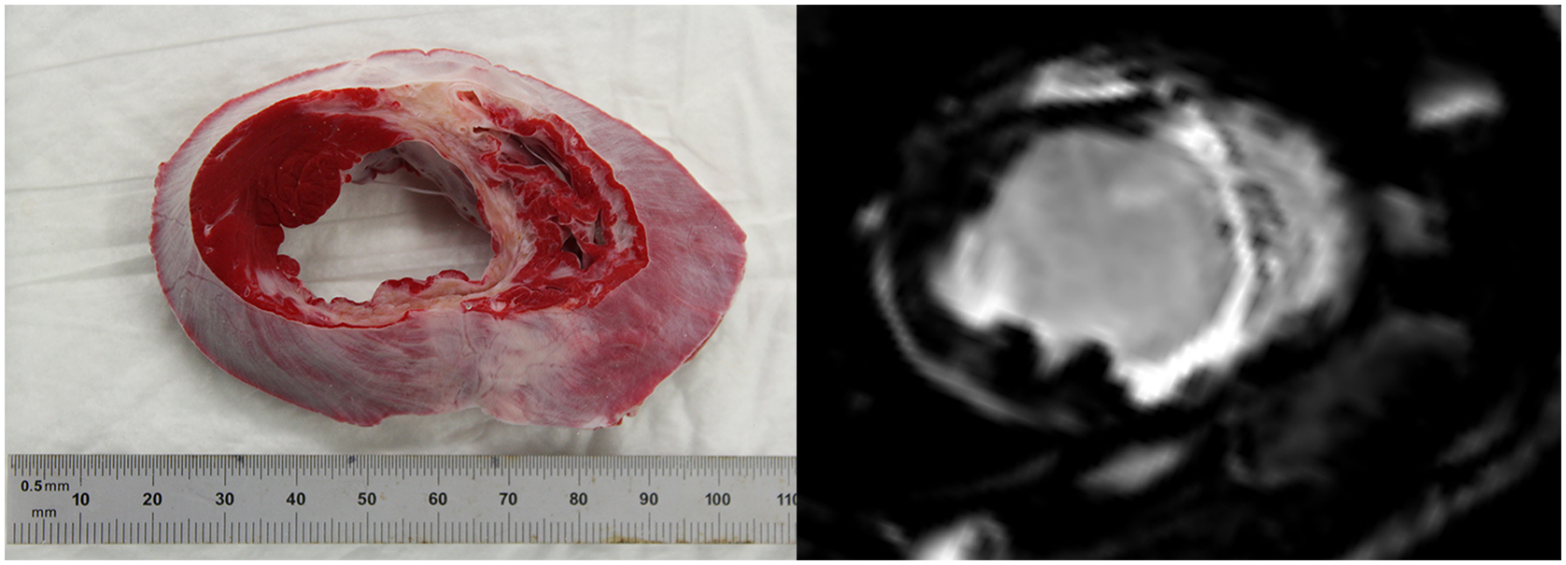
Infarct size. Representative pictures of TTC stained heart slice and LGE image of the same animal. Mean (±SD) infarct size of CMPC and placebo animals were 14.6 ± 2,5% and 13.8 ± 3.9% of the left ventricle based on TTC staining and 17.5 ± 3.8 and 18.0 ± 4.5 based on LGE imaging.

For LGE, infarct size was 17.5 ± 3.8% and 18.0 ± 4.5% of the left ventricular mass for cell treated and placebo animals respectively at follow up. For TTC, infarct size in the cell treated group was 14.6 ± 2.5% of the left ventricle, and 13.8 ± 3.9 in the control group. There was no statistical difference in infarct size between both groups at termination of follow-up, for both modalities. (Fig 5)

### Histology

**Fig 6:**
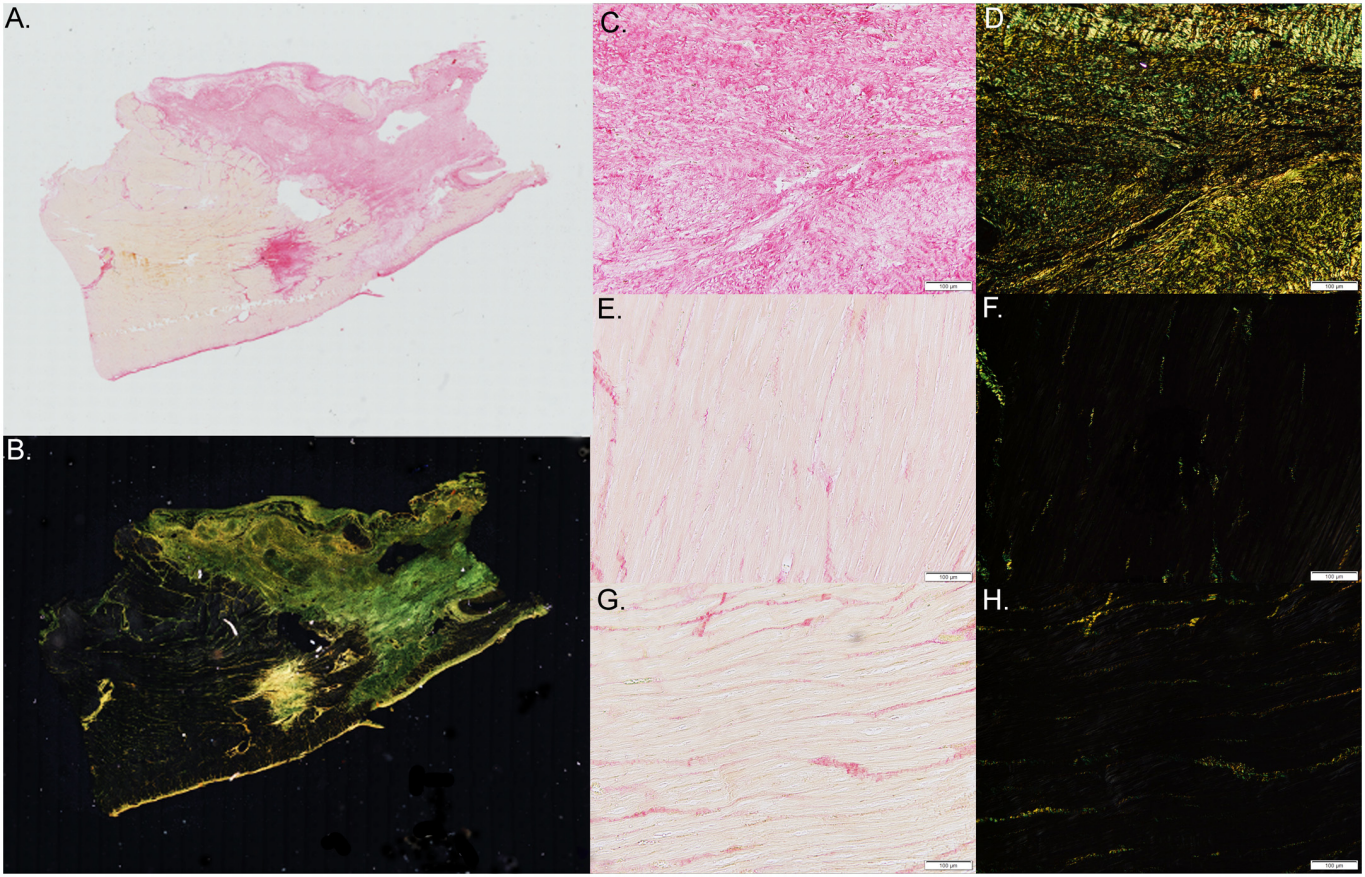
Fibrosis. Representative picture of a picrosirius red stained borderzone slide (A. bright field, B. polarized light) and a higher magnification of a picrosirius red stained slides isolated from the infarct area (C,D), borderzone (E,F) and remote area (G,H) of the left ventricle (bright field C,E,G, polarized light (D,F,H).

**Fig 7:**
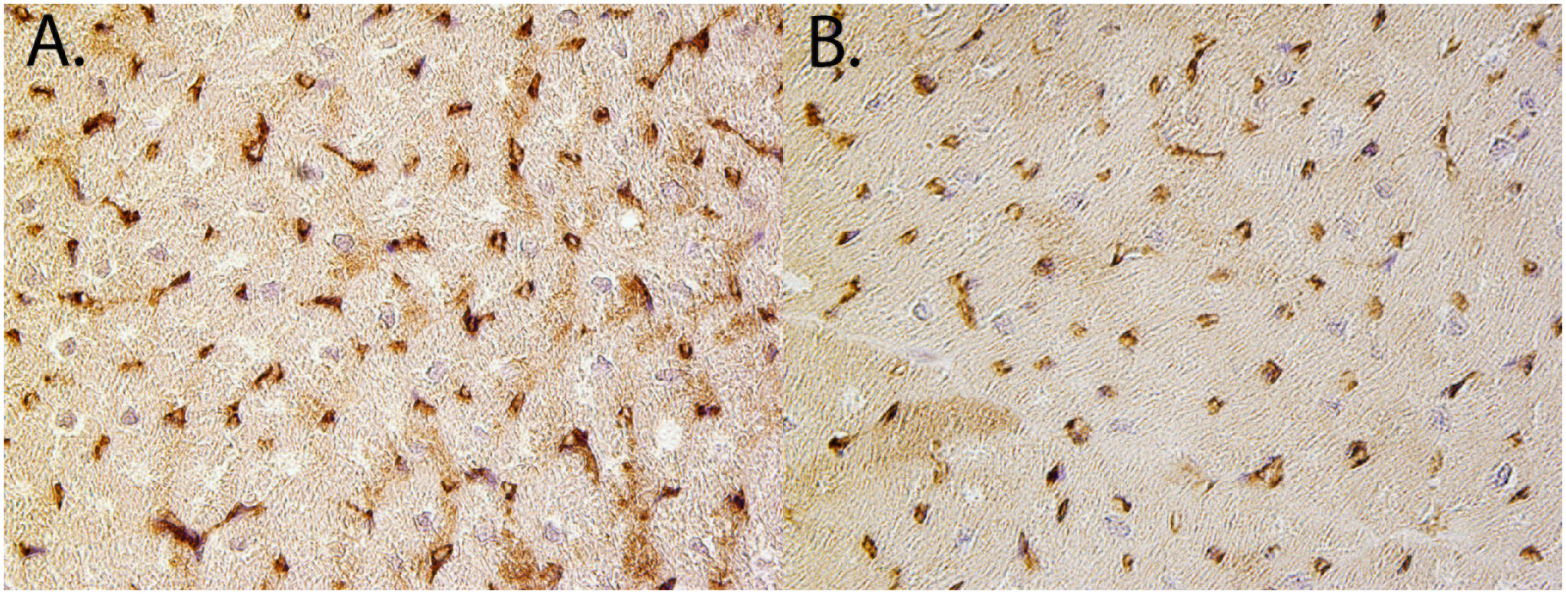
Vascular density. Respresentative picutures of Lectin staining of the borderzone of a CMCP treated animals (A) and a placebo animal (B)

For fibrosis, there were no differences between both groups. A significant higher vascular density was observed in the placebo group compared to the hCMPC animals. (Table 2, Figs 6 and 7)

**Table 2:** Fibrosis.

	CMPC (n = 8)	Placebo (n = 8)
% Fibrosis		
Infarct	39.1 ± 12.2	40.0 ± 16.0
Border	1.6 ± 1.2	2.9 ± 2.3
Remote	6.5 ± 7.8	2.8 ± 2.3
Grey value		
Infarct	27.0 ± 8.9	28.5 ± 12.7
Border	1.2 ± 0.7	2.0 ± 1.4
Remote	4.3 ± 4.3	2.6 ± 2.2
Vascular density	33.9 ± 10.1	48.3 ± 10.1 *

* p = 0.01 between CMPC and placebo.

### Cyclosporin

Mean CsA levels at follow up per animal is provided in S4 Table. *In vitro* assays showed that CsA did not affect functioning of CMPCs with regard to migration capacity, angiogenesis and growth factor secretion. See S1 File and S2–S4 Figs for supporting information and the results of the *in vitro* assays.

### QPCR

**Fig 8:**
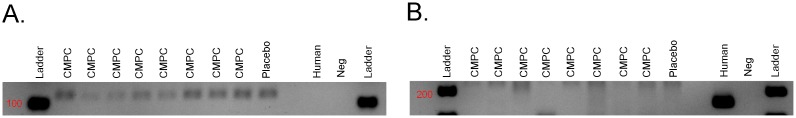
QPCR. A. QPCR shows presence of genomic pig DNA (Gapdh) in all pig tissues treated with CMPC or placebo and absence of pig DNA in the sample containing human CMPCs (Human) and the negative control (Neg) containing H2O used for PCR. B. Human genomic DNA (HLA-DMA) was detected in the sample containing human CMPCs (Human), but was absent in all the pig tissues (CMPC and Placebo) and the negative control (Neg) containing H2O used for PCR

Quantitative PCR showed absence of human DNA in all pig tissue samples. HLA was successfully amplified in the positive control (samples containing fhCMPCs). Pig DNA (Gapdh) was present in all samples containing pig tissue. Blotting of the samples after running them on gel confirmed absence of human material in the pig tissue samples. (Fig 8)

## Discussion

In this pre-clinical model of chronic MI, we show that xenotransplantation via intracoronary infusion of fetal hCMPCs is feasible and safe, but we did not find a benefit in the current model on cardiac function, infarct size, fibrosis and vascular density. The non-significant difference in EF between both groups of 2.3% measured by the golden standard MRI is lower than the effect size of 7.5% as documented in a recent meta-analysis and used for power calculation.

We believe that these data in the field of cardiac regeneration are of great importance. It complements the preclinical evidence needed for successful translation of cardiac regeneration by cardiac cell therapy from bench to bedside. We present a solid and sound piece of evidence since this study is conducted by clinical standards in terms of blinding, randomization and outcome assessment.

### Preclinical studies of cardiac cell therapy

The neutral results presented in the current study, are partly in line with other large animal studies with cardiac stem cells for MI (Table 3). Over the last decade, five different cardiac stem cell sources have been identified[11] and two of them have been extensively tested in large animal studies. Clonogenic, multipotent and self-renewing cardiac stem cells, based on the C-kit epitope (CSCs)[12,13] and cluster-forming cells, positive for C-kit and Sca-1, called cardiosphere derived cells (CDCs) [14] were used in these studies. Both cell types have gone through the process of pre-clinical investigation and progressed from bench to bedside testing. In a recent study, expanded cells upon different cardiac progenitor cell isolation procedures were compared (including, c-kit+, cardiospheres, and sca1+ cells), and it was suggested that these cells have highly similar transcriptional profiles.[15] Therefore, it is important to closely look at particular study design aspects of the study to look for possible explanations

**Table 3:** Overview of studies of cardiac derived stem cells in pig models.

	N	Route	Source	Immuno suppression	Dose	Timing	Follow up	Result
**CDC**								
**Johnston et al. Circulation 2009** [17]	17	IC	Autologous	NA	1,0E+07	4 wk	8 wk	No EF or IS improvement
**Lee et al. JACC 2011** [18]	29	IM	Autologous	NA	1,0E+07	4 wk	8 wk	No functional improvement on echo. EF improved on LV angiography
**Malliaras et al. Circulation 2013** [19]	10	IC	Allogeneic	No	1,25E+07	2–3 wk	8 wk	EF preservation, IS reduction
**Kanazawa et al.**[20] **Circ. Heart Fail. 2015**	14	IC	Allogeneic	No	8,5-9E+06	30 mins	2 days	No difference in EF, IS reduction
**Yee et al**. *[21] **PLoS ONE 2014**	22	TE	Allogeneic	No	1,5E+08	8 wk	8 wk	No functional improvement, IS reduction
**CSC**								
**Williams et al. Circulation 2013** [22]	10	IM	Human	Cyclosporin	1,0E+06	14 days	1 mnth	Restored EF, IS reduction
**Bolli et al. Circulation 2013** [23]	21	IC	Autologous	NA	5,0E+05	3–4 mnth	1 mnth	Improved EF, IS reduction

IM intramyocardial injections, IC intracoronary infusion, TE transendocardial injections, EF ejection fraction, IS infarct size.

* used cardiospheres instead of CDCs.

### Study design considerations

A multitude of parameters in the research field of cell therapy for cardiac repair must be considered when conducting pre-clinical or clinical studies, like timing of therapy, administration route, cell source, cell number, etc. In a meta-analysis including all large animal studies on cell therapy for MI, none of these parameters showed significant impact on effect size.[16] Additionally, no ruling about these issues was provided based on previous CSC studies (Table 3). Therefore, we made upfront decisions about all these variables based on experience and clinical relevance.

Treatment of acute myocardial infarction is greatly improved over the last decades, but patients surviving the initial episode often suffer from chronic ischemic heart failure. The current animal model was designed to represent this group. A direct comparison of efficacy of stem cells in acute versus chronic ischemic heart disease has never been performed, but safety is at least similar in acute and chronic patients.[24] Table 3 shows negative and positive results of cardiac cell therapy in both acute and chronic settings.

We administered cells via intracoronary infusion, which is considered as efficient as other routes with regard to cell retention [25–27] and functional improvement [28,29] but is less invasive then intramyocardial injections and less time consuming compared to transendocardial delivery.

We investigated efficacy of infusion of 10 million cells. Table 3 show both positive and neutral results from CDCs in comparable doses. For CSCs, promising results are found by infusion of 0.5 and 1 million cells in smaller pigs (1.5 ± 0.8kg and 35–40 kg respectively, compared to 68.5 ± 5.4 kg in the current study).[22,23] Roughly, we applied 4 times higher indexed doses (37.000/kg versus 140.000/kg) of cells. These data show that relative under-dosing was certainly not anticipated for in the present study.

### Xenogenicity

The impact of xenogenicity is important since human cells were administered in a porcine model. Alloreactivity (and xenoreactivity) depends on foreign peptide presentation by the major histocompatibility complex (MHC) on antigen presenting cells and detection by T cells.[30] In clinical practice, alloreactivity is suppressed by T cell suppressors like CsA, and this strategy was applied accordingly in the present study.

Next to immunosuppression, the effect of CsA includes prevention of apoptosis [31] and protection of the myocardium in the setting of acute myocardial infarction.[32]. Since CsA was administered at 4 weeks after MI until the end of follow-up, we believe it did not affect the outcome by that cause. Adequate dosing by oral administration of CsA is safe in pigs[33], but not much is known about the pharmacodynamics. We did not observe differences in serum levels of leukocytes, nor on renal function between hCMPC and placebo treated animals 30 minutes and 4 weeks after treatment, all receiving CsA. Also, we did not find a correlation between serum CsA levels at follow up and effect size.

There is no consensus about the effect of CsA on stem cells *in vitro* and *in vivo*.[33] In this respect, we tested the effect of different levels of CsA on migration, angiogenesis and growth factor expression of hCMPCs *in vitro* (See S1 File). No significant effect of CsA was noted. Taken together, we assume that our results are not confounded by the chosen CsA regimen.

Despite adequate immunosuppression, we anticipated on low cell retention rates. Based on a study by our own lab, and several other studies, we know that short term cell retention is around 10–15% irrespective of the administration route.[17–19] Long term cell retention is expected to be even lower. Despite these discouraging numbers, administration of cells does improve cardiac function in almost all clinical and preclinical studies.[16,34] This supports the proposed working mechanism by endogenous signaling and the redundancy of (long-term) cell retention to establish effect.[35,36] As anticipated, no human cells could be traced in the small pieces of tissue that we collected from the borderzone area of the pig hearts 4 weeks after therapy. However, presence of human cells in the rest of the heart cannot be excluded with complete certainty.

### Internal and external validity

Internal validity has major impact on effect size, as it is known that blinded and randomized studies produce smaller effect sizes compared to non-blinded and non-randomized reports.[37] Based on clinical standards, our pre-clinical study has been performed in a randomized, blinded (both for intervention and outcome assessment) and placebo-controlled manner.

The animal model is a surrogate for human disease. We included large pigs (68.5 ± 5.4 kg), as the heart corresponds with human (coronary) anatomy and size, to increase external validity (the translatability towards other models or populations). However, age and co-morbidity are not accounted for in this model.

### Preclinical studies of non-cardiac cell therapy for cardiac regeneration

Next to cardiac tissue derived cell types, embryonic stem cell derived- and induced pluripotent derived cardiomyocytes (ESC-CMs and IPCS-CMc) may hold promise for cardiac regenerative therapy. The first results of transplantation of human ESC-CMs in a large animal model of non-human primates are promising since hESC-CMs showed to remuscularize the heart.[38] In the four treated animals, grafts are vascularized and electromechanically coupled. The major drawback of this this small open-label non-randomized study is the observed ventricular arrhythmias. Next, transplantation of human IPSC-CMs combined with IPCS derived endothelial cells and smooth muscle cells into the myocardium of a porcine model of acute MI shows engraftment of cells and development into functioning cardiomyocytes accompanied by improvement of left ventricular function and infarct size.[39]

These two studies show the potential of non-cardiac derived cardiac-cell therapy. Further investigation of cardiac regeneration by these cell sources is needed for safe and successful clinical translation.

Our standardized way of infarct creation caused a decrease in EF of 8.7% based on PV-loop measurements, despite high TnI levels and a mean infarct size of 17.8%. It is known from clinical studies that patients with a low baseline EF benefit most from cell therapy.[40,41] In retrospect, the anticipated effect size of 7.5% (difference in EF at follow up between cell and placebo treated animals on MRI) might have been too high. Power calculation was based on difference in EF at follow up, measured by MRI and not for the other functional measurement modalities (echocardiography and PV-loop), which might thereby be underpowered to report significant differences.

Finally it has to be acknowledged that the Sca-1 antigen (stem cell antigen 1) is well characterized in mice. In 2003 cardiac stem cells were isolated from mouse hearts using anti-sca-1 antibodies. These cells showed to be multipotent and self-renewing.[42] Although the human equivalent of the Sca-1 epitope is unknown, anti-Sca-1 antibodies are successfully used to reproducibly isolate stem cells from fetal and adult human hearts.[4] These cells express Isl-1, C-kit, CD31, GATA-4 and Nkx2.5, but no cActin and cTroponin and were therefore called cardiomyocyte progenitor cells.[2, 4,] Furthermore, despite different isolation procedures, CMPCs turned out to share high similarity in gene expression profiles with other cardiac cell lines including CSCs and cardiosphere derived cells.[15]

## Conclusion

In this randomized, blinded placebo controlled preclinical study, xenotransplantation via intracoronary infusion of fetal human CMPCs in a pig model of chronic I/R injury appeared to be feasible and safe, but showed no significant improvement with regard to cardiac function or infarct size.

## Supporting Information

S1 Fig fhCMPCs in culture. fhCMPCs in culture, attached to the survace (A.) and in suspension (B.)
(TIF)

S2 Fig Quantification of cell migration. (TIF)

S3 Fig Sprouting matrigel assay. A. bar plot of angiogenesis parameters. B. representative pictures of matrigel assays for all 4 conditions.
(TIF)

S4 Fig Array map and pictures of the array of 4 conditions at three different intensities. Positive control meaning different cell line without Cyclosporin
(TIFF)

S1 File Supporting information. (DOC)

S1 Table Angiogenesis. (DOCX)

S2 Table Functional outcome measured by PV loop. EDV End diastolic volume, ESV End systolic volume, EF Left ventricular ejection fraction, SW Stroke work, ESPRV End systolic pressure volume relationship, EDPVR End diastolic pressure volume relationship, dPdT+ maximum pressure rise, dPdT- Minimum pressure rise, V0 Theoretical volume at zero pressure. * p = 0.03 Cell treated animals compared to placebo. For EDV, ESV and EF n = 7 for cell treated animals, n = 8 for placebo treated animals. For SW, ESPRV, EDPVR, dPdT+, dPdT-, V0 and Tau n = 6 per group.
(DOCX)

S3 Table Functional outcomes measured by echocardiography. sWT Septal wall thickening, FS Fractional shortening, FAS Fractional area shortening at levels of the mitral valve (mitral) papillary muscle (pap), apex and mean of three levels (mean). N = 8 per group. *p = 0.08 for cell treated animals compared to placebo.
(DOCX)

S4 Table Individual Ciclosporin levels at end of follow up. (DOCX)

S1 Video Long axis echocardiography. Representative long axis view echocardiography, 4 weeks after myocardial infaction (MI), right before CMPC/placebo infusion. Thinning and akinesia of the septal apical wall due to MI can be appreciated.
(AVI)

S2 Video Short axis echocardiography. Representative short axis view echocardiography at the level of the apex, 4 weeks after myocardial infaction (MI), right before CMPC/placebo infusion. Thinning and akinesia of the septal wall due to MI can be appreciated.
(AVI)
