# Supplementary figures and images for: Xenotransplantation of Human Cardiomyocyte Progenitor Cells Does Not Improve Cardiac Function in a Porcine Model of Chronic Ischemic Heart Failure. Results from a Randomized, Blinded, Placebo Controlled Trial
Source: PLoS One. 2015 Dec 17;10(12):e0143953. doi: 10.1371/journal.pone.0143953 (PMC4683045; doi:10.1371/journal.pone.0143953)

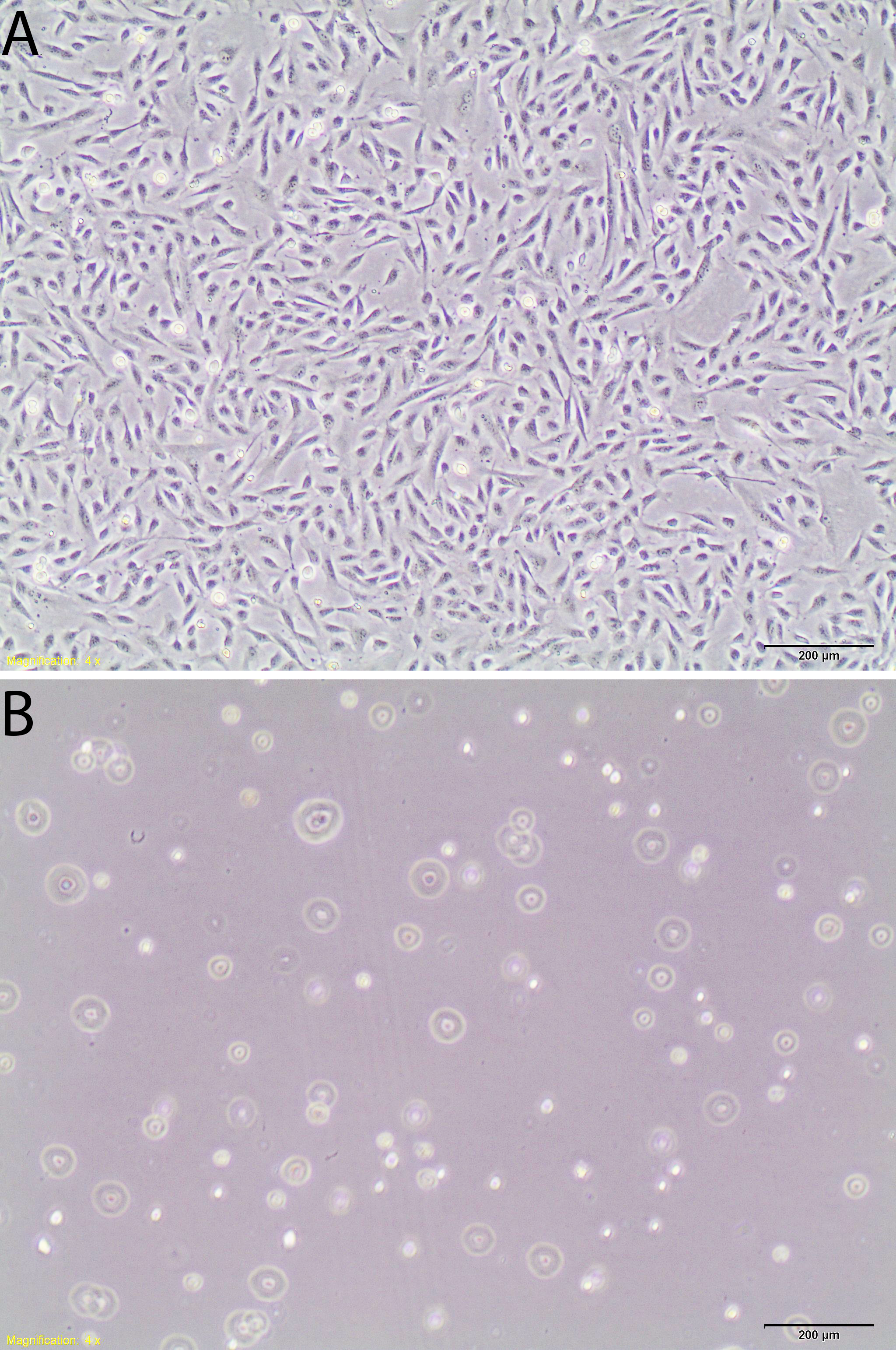

Supplement: S1 Fig — fhCMPCs in culture, attached to the survace (A.) and in suspension (B.) (TIF) [file pone.0143953.s001.tif]

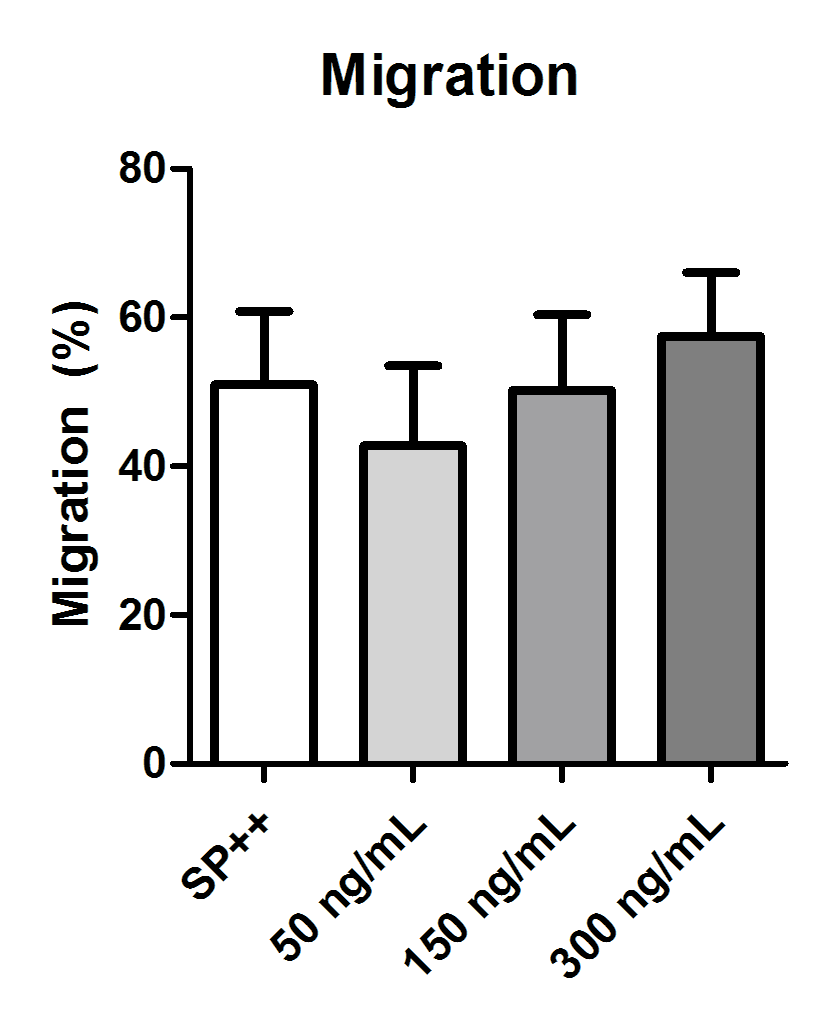

Supplement: S2 Fig — (TIF) [file pone.0143953.s002.tif]

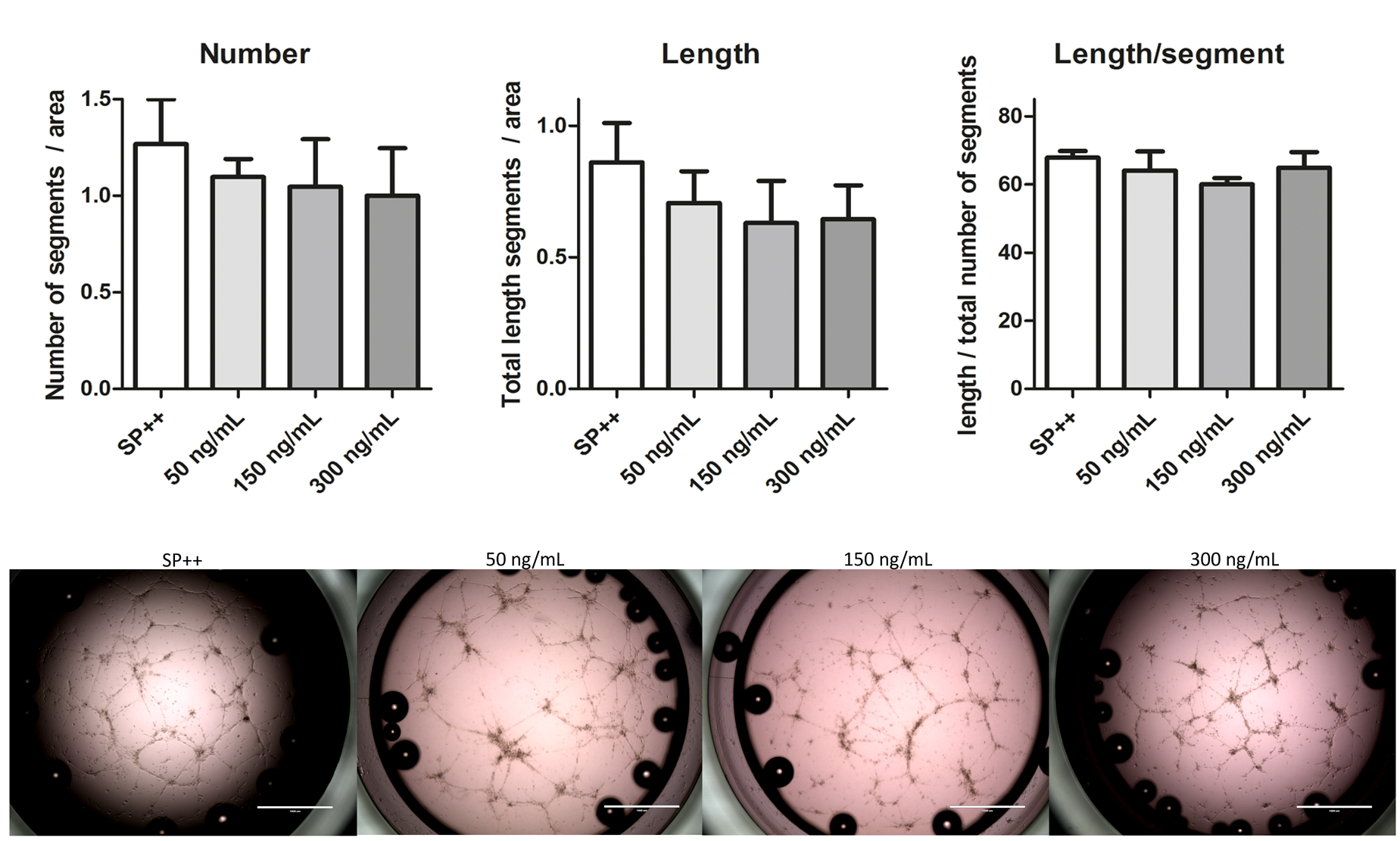

Supplement: S3 Fig — A. bar plot of angiogenesis parameters. B. representative pictures of matrigel assays for all 4 conditions. (TIF) [file pone.0143953.s003.tif]

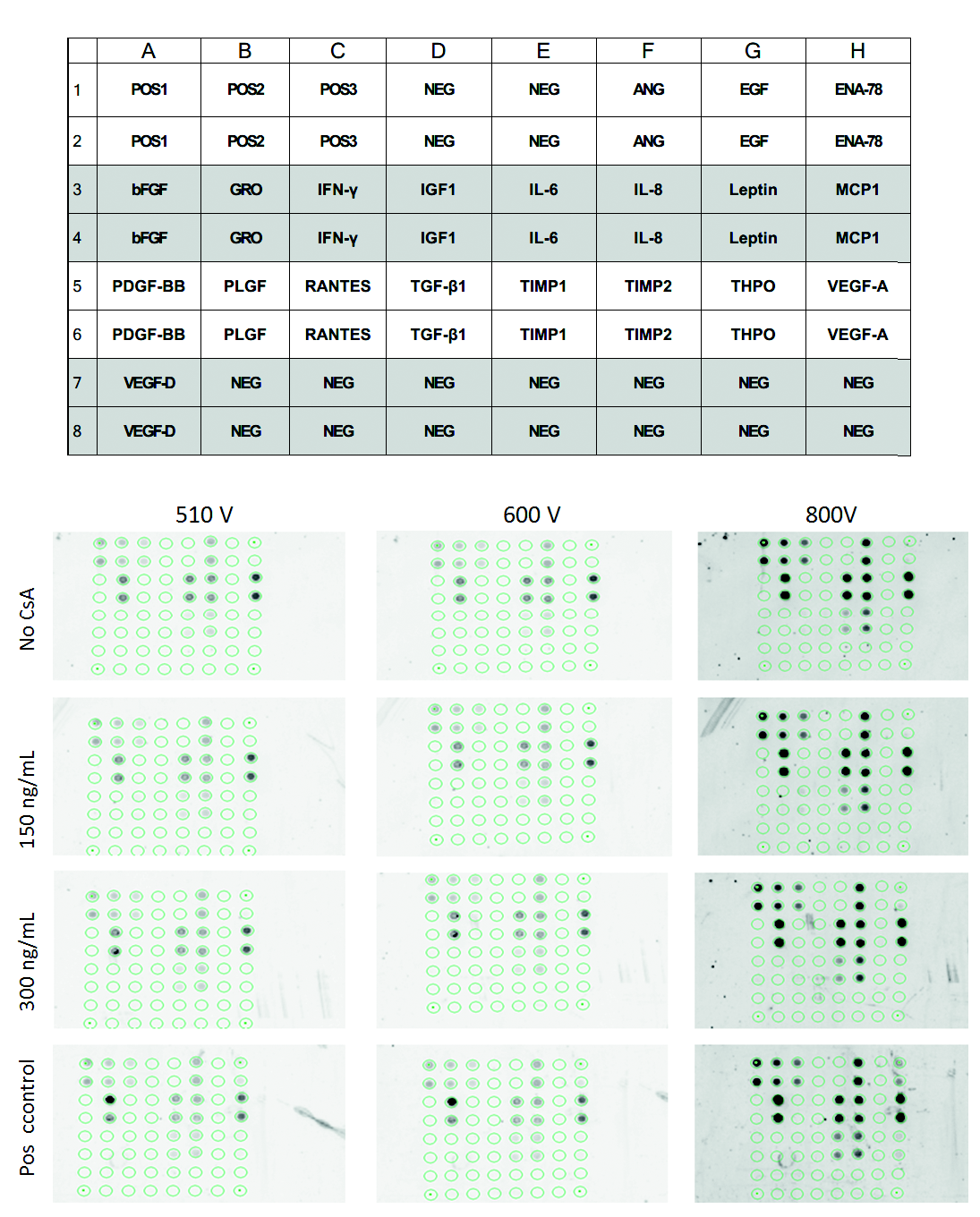

Supplement: S4 Fig — Positive control meaning different cell line without Cyclosporin (TIFF) [file pone.0143953.s004.tiff]
