# Supplementary material for: Xenotransplantation of Human Cardiomyocyte Progenitor Cells Does Not Improve Cardiac Function in a Porcine Model of Chronic Ischemic Heart Failure. Results from a Randomized, Blinded, Placebo Controlled Trial
Source: PLoS One. 2015 Dec 17;10(12):e0143953. doi: 10.1371/journal.pone.0143953 (PMC4683045; doi:10.1371/journal.pone.0143953)
Supplement: S1 File — (DOC) [file pone.0143953.s005.doc]

**Supporting methods**

Histology

For staining, slides were deparafinated by 2x10minutes ultraclear, 2x5 minutes 99% EtOH, 2x5 minutes 96% EtOH and 5 minutes 7% EtOH. Slides were washed three times with dH2O for 5 minutes. For picrosirius staining, all slides were stained at once for 30 minutes in filtered picrosirus red. Slides were washed twice 0.2N HCl, then in dH2O for 5 minutes and dehydrated (quick 70% EtOH, 2x quick in 96% EtOH, 2x 5 minutes 99% EtOH, 2x10 minutes Ultraclear), mounted with entallan and covered with coverslips.

For lectin, deparafinated slides were incubated with 3% H2O2 for 20 minutes and with 1% BSA in PBS for 15 minutes. Slides were incubated over night with Lectin (1:50 in PBS). After washing twice with PBS, slides were stained with DAB+ chromogen in DAB+ substrate buffer (Dako K3467) for a maximum of 4 minutes. Slides were washed with running tap water, dehydrated as above, mounted with entellan and covered with coverslips.

Cyclosporin assays

Migration assay

A migration scratch assay was performed to compare migratory capacity. CMPCs were plated in 0.1% gelatin coated 6 well plates in medium with additional CsA in three concentrations. At 90% confluency a scratch was placed vertically in the middle of the well using a pipet tip, after which medium was changed and replaced with either fresh medium or medium containing CsA. Pictures of the initial scratches were immediately taken. After 3 and 5 hours new pictures were taken of the scratched area. Analysis of migration capacity was performed using the ImageJ software.

Sprouting matrigel assay

Angiogenesis μ-slides (Ibidi) were used to quantify matrigel tube formation. Slide chambers were coated with 10 μl ECMatrixTM (Millipore). CMPCs were seeded on the matrigel in 50 μl, with or without CsA. Tube formation was imaged after 13 hours and quantified using the Angioquant software in Matlab.

Growth factor antibody array

Conditioned medium was collected from the CMPCs after 9 days of culture in low serum medium without any additional growth factors, with or without CsA. Human Angiogenesis Array G1 (RayBiotech) was performed on the conditioned medium according to the provided instructions. Three conditions were tested; normal medium, 150 ng/mL and 300 ng/mL. Next, we tested culture medium from a different CMPC cell line as a positive control.

**Results**

Migration of CMPC was the same for all conditions (Supporting figure 1). The migration was 51.0 ± 9.8 % for the normal medium and 42.8 ± 10.7%, 50.2 ± 10.1% and 57.5 ± 8.5% in the presence of CsA (50ng/mL, 150 ng/mL and 300 ng/mL respectively) (p=0.95)

For angiogenesis, the number of segments per area, total length per area and length per segment were calculated. For all 3 measures, no difference exists between conditions (Supporting table 3, figure 2).

Conditioned medium of CMPCs cultured for 9 days contained Angiogenin (ANG), CXCL 1, 2&3 (GRO), Il-6, Il-8, CCL2 (MCP1), CCL5 (RANTES) and metallopeptidase inhibitor 1 (TIMP1). Presence of CsA did not affect growth factor secretion by CMPCs (Supporting figure 3).
