# Supplementary material for: Xenotransplantation of Human Cardiomyocyte Progenitor Cells Does Not Improve Cardiac Function in a Porcine Model of Chronic Ischemic Heart Failure. Results from a Randomized, Blinded, Placebo Controlled Trial
Source: PLoS One. 2015 Dec 17;10(12):e0143953. doi: 10.1371/journal.pone.0143953 (PMC4683045; doi:10.1371/journal.pone.0143953)
Supplement: S1 Table — (DOCX) [file pone.0143953.s006.docx]

Supporting table 1. Angiogenesis

|  | **medium** | **50 µg/L** | **150 µg/L** | **300 µg/L** | **P-value** |
| --- | --- | --- | --- | --- | --- |
| **Total number/area** | 1.26 ± 0.23 | 1.10 ± 0.09 | 1.05 ± 0.25 | 1.00 ± 0.25 | 0.49 |
| **Total length/area** | 86.0 ± 15.0 | 70.6 ± 12.0 | 63.1 ± 15.9 | 64.5 ± 12.8 | 0.25 |
| **Length/number** | 67.9 ± 1.8 | 64.1 ± 5.6 | 60.1 ± 1.8 | 64.9 ± 4.6 | 0.18 |
