# Supplementary material for: Xenotransplantation of Human Cardiomyocyte Progenitor Cells Does Not Improve Cardiac Function in a Porcine Model of Chronic Ischemic Heart Failure. Results from a Randomized, Blinded, Placebo Controlled Trial
Source: PLoS One. 2015 Dec 17;10(12):e0143953. doi: 10.1371/journal.pone.0143953 (PMC4683045; doi:10.1371/journal.pone.0143953)
Supplement: S2 Table — EDV End diastolic volume, ESV End systolic volume, EF Left ventricular ejection fraction, SW Stroke work, ESPRV End systolic pressure volume relationship, EDPVR End diastolic pressure volume relationship, dPdT+ maximum pressure rise, dPdT- Minimum pressure rise, V0 Theoretical volume at zero pressure. * p = 0.03 Cell treated animals compared to placebo. For EDV, ESV and EF n = 7 for cell treated animals, n = 8 for placebo treated animals. For SW, ESPRV, EDPVR, dPdT+, dPdT-, V0 and Tau n = 6 per group. (DOCX) [file pone.0143953.s007.docx]

Supporting table 2. Functional outcome measured by PV loop

|  |  | **Cell** |  |  | **Placebo** |  |
| --- | --- | --- | --- | --- | --- | --- |
|  | **Baseline** | **Pre-infusion** | **Follow up** | **Baseline** | **Pre-infusion** | **Follow up** |
| **EDV (ml)** | 185.1 ± 43.7 | 237.1 ± 51.4 | 275.5 ± 64.1 | 188.1 ± 34.3 | 213.9 ± 51.3 | 274.9 ± 51.0 |
| **ESV (ml)** | 85.6 ± 33.3 | 141.2 ± 57.0 | 157.0 ± 38.3 | 84.9 ± 22.9 | 112.5 ± 39.9 | 154.2 ± 34.3 |
| **EF (%)** | 54.6 ± 9.8 | 43.5 ± 9.8 | 42.7 ± 6.0 | 55.0 ± 8.3 | 48.4 ± 9.3 | 43.7 ± 9.7 |
| **SW** | 1.3 ± 0.4 | 0.9 ± 0.8 | 1.6 ± 0.8 | 1.3 ± 0.4 | 0.9 ±0.7 | 1.7 ± 0.4 |
| **ESPVR** | 1.3 ± 0.6 | 1.2 ± 0.7 | 1.9 ± 1.8 | 1.3 ± 0.6 | 1.5 ± 0.9 | 1.2 ± 0.7 |
| **EDPVR** | 0.022 ± 0.020 | 0.008 ± 0.007 | 0.007 ± 0.004 | 0.017 ± 0.013 | 0.006 ± 0.004 | 0.004 ± 0.002* |
| **dPdT+** | 1559.3 ± 196.3 | 1483.9 ± 414.0 | 1667.1 ± 324.9 | 1544 ± 210.4 | 1575.9 ± 182.3 | 1316.9 ± 97.9 |
| **dPdT-** | -1231.3 ± 195.9 | -1213.1 ± 379.4 | -1305.5 ± 274.7 | 1388.4 ± 224.7 | -1223.0 ± 248.8 | -1077.7 ± 269.2 |
| **V0** | -49.9 ± 71.8 | -14.4 ± 71.5 | -6.9 ± 139.0 | -34.7 ± 49.0 | -14.5 ± 57.5 | -26.8 ± 54.3 |
| **Tau** | 45.8 ± 9.6 | 55.2 ± 22.4 | 53.4 ± 10.2 | 40.2 ± 8.4 | 48.4 ± 7.5 | 60.6 ± 14.8 |
