# Supplementary material for: Xenotransplantation of Human Cardiomyocyte Progenitor Cells Does Not Improve Cardiac Function in a Porcine Model of Chronic Ischemic Heart Failure. Results from a Randomized, Blinded, Placebo Controlled Trial
Source: PLoS One. 2015 Dec 17;10(12):e0143953. doi: 10.1371/journal.pone.0143953 (PMC4683045; doi:10.1371/journal.pone.0143953)
Supplement: S3 Table — sWT Septal wall thickening, FS Fractional shortening, FAS Fractional area shortening at levels of the mitral valve (mitral) papillary muscle (pap), apex and mean of three levels (mean). N = 8 per group. *p = 0.08 for cell treated animals compared to placebo. (DOCX) [file pone.0143953.s008.docx]

Supporting table 3. Functional outcomes measured by echocardiography

|  |  | **Cell** |  |  | **Placebo** |  |
| --- | --- | --- | --- | --- | --- | --- |
|  | Baseline t=0  (n=8) | Pre-infusion  (n=8) | Follow up  (n=8) | Baseline t=0  (n=8) | Pre-infusion  (n=8) | Follow up  (n=8) |
| **sWT mitral** | 0,18 ± 0.12 | 0.23 ± 0.12 | 0.27 ± 0.20 | 0.29 ± 0.20 | 0.28 ± 0.11 | 0.14 ± 0.16 |
| **sWT pap** | 0.36 ± 0.13 | 0.37 ± 0.15 | 0.35 ± 0.24 | 0.39 ± 0.15 | 0.35 ± 0.32 | 0.42 ± 0.41 |
| **sWT apex** | 0.58 ± 0.26 | 0.17 ±0.24 | 0.22 ± 0.27 | 0.47 ± 0.14 | 0.18 ± 0.21 | 0.23 ± 0.32 |
| **sWT mean** | 0.37 ± 0.13 | 0.26 ± 0.10 | 0.28 ± 0.13 | 0.38 ± 0.14 | 0.27 ± 0.16 | 0.26 ± 0.21 |
| **FS mitral** | 0.23 ± 0.06 | 0.29 ± 0.05 | 0.29 ± 0.05 | 0.28 ± 0.05 | 0.30 ± 0.04 | 0.27 ± 0.06 |
| **FS pap** | 0.23 ± 0.05 | 0.28 ± 0.05 | 0.27 ± 0.04 | 0.24 ± 0.05 | 0.30 ± 0.04 | 0.25 ± 0.06 |
| **FS apex** | 0.24 ± 0.08 | 0.20 ± 0.06 | 0.19 ± 0.08 | 0.24 ± 0.05 | 0.19 ± 0.06 | 0.18 ± 0.05 |
| **FS mean** | 0.23 ± 0.03 | 0.25 ± 0.04 | 0.25 ± 0.04 | 0.26 ± 0.05 | 0.24 ± 0.05 | 0.23 ± 0.04 |
| **FAS mitral** | 0.49 ± 0.09 | 0.55 ± 0.05 | 0.57 ± 0.05 | 0.54 ± 0.08 | 0.55 ± 0.07 | 0.53 ± 0.07 |
| **FAS pap** | 0.46 ± 0.08 | 0.47 ± 0.06 | 0.49 ± 0.08 | 0.48 ± 0.09 | 0.478 ± 0.12 | 0.44 ± 0.07 |
| **FAS apex** | 0.45 ± 0.07 | 0.40 ± 0.07 | 0.42 ± 0.08 | 0.47 ± 0.12 | 0.37 ± 0.08 | 0.36 ± 0.05 |
| **FAS mean** | 0.47 ± 0.03 | 0.42 ± 0.17 | 0.49 ± 0.06* | 0.49 ± 0.08 | 0.47 ± 0.07 | 0.44 ± 0.04* |
