# Supplementary material for: Xenotransplantation of Human Cardiomyocyte Progenitor Cells Does Not Improve Cardiac Function in a Porcine Model of Chronic Ischemic Heart Failure. Results from a Randomized, Blinded, Placebo Controlled Trial
Source: PLoS One. 2015 Dec 17;10(12):e0143953. doi: 10.1371/journal.pone.0143953 (PMC4683045; doi:10.1371/journal.pone.0143953)
Supplement: S4 Table — (DOCX) [file pone.0143953.s009.docx]

| Control | | CMPC treated | |
| --- | --- | --- | --- |
| Pig | CsA level | Pig | CsA level |
| 3 | 160 ng/L | 1 | 30 ng/L |
| 4 | 60 ng/L | 2 | 100 ng/L |
| 9 | 140 ng/L | 7 | 90 ng/L |
| 12 | 40 ng/L | 8 | 110 ng/L |
| 13 | 140 ng/L | 10 | 30 ng/L |
| 15 | 170 ng/L | 11 | 30 ng/L |
| 16 | 80 ng/L | 14 | 30 ng/L |
| 19 | 280 ng/L | 18 | 110 ng/L |
